# Evaluation of Serum Levels of Inflammation, Fibrinolysis and Oxidative Stress Markers in Coronary Artery Disease Prediction: A Cross-Sectional Study

**DOI:** 10.5935/abc.20190159

**Published:** 2019-10

**Authors:** Sakhavat Abolhasani, Shahnam Valizadeh Shahbazloo, Hossein Mozafar Saadati, Neda Mahmoodi, Nafiseh Khanbabaei

**Affiliations:** 1 Department of Clinical Biochemistry - Student Research Committee - School of Medicine - Shahid Beheshti University of Medical Sciences, Tehran, Iran; 2 Department of Clinical Biochemistry - Faculty of Medicine - Tabriz University of Medical Sciences, Tabriz, Iran; 3 Department of Epidemiology - School of Public Health and safety - Shahid Beheshti University of Medical Sciences, Tehran, Iran

**Keywords:** Coronary Artery Disease, Biomarkers, Inflammation Fibrinolysis, Oxidative Stress, Sialic Acids, Vitronectin

## Abstract

**Background:**

Coronary Artery Disease (CAD) has long been recognized as a global health issue. Inflammation, Fibrinolysis and Oxidative Stress play an important role in the disruption of plaques leading to CAD. Markers that reflect this pathophysiologic mechanism may have prognostic value.

**Objective:**

To estimate the serum concentrations of high-sensitivity C-reactive protein (hs-CRP), sialic acid (SA), vitronectin (VN), plasminogen activator inhibitor-1 (PAI-1), oxidized low density lipoprotein (OX-LDL) and malondialdehyde (MDA) with significant prognostic value in patients with CAD.

**Methods:**

The markers included, hs-CRP, SA, VN, PAI-1, OX-LDL and MDA, were compared between 160 angiographically diagnosed CAD patients and 20 age- and sex-matched healthy individuals. The subjects were divided into 4 groups according to angiography results, and association between all risk factors of CAD was studied. Serum levels of SA, VN, PAI-1, and OX-LDL were measured by enzyme-linked immunosorbent assay (ELISA); MDA was measured based on reaction with thiobarbituric acid (TBA); and hs-CRP level was estimated by immunoturbidimetry using a commercial kit. The diagnostic value of these variables was further assessed by ROC curve analysis. Multiple logistic regression was used to evaluate the diagnostic power of the combination. Furthermore, p < 0.05 was considered as significant.

**Results:**

Serum levels of hs-CRP, SA, VN, PAI-1, and OX-LDL were significantly higher in patient groups compared to control group (p < 0.001). Using both normal and CAD patients as subjects, ROC analysis was performed. The cutoff for OX-LDL, MDA, PAI-1, VN, hs-CRP and SA was 2.67 (ug/mL), 5.49 (mmol/mL), 67 (ng/mL), 254 (ng/mL), 3.4 (mg/dL), 7/89 (mg/dL), respectively. Eventually, the complete diagnostic efficacy was classified as: SA, hs-CRP, PAI-1, OX-LDL, MDA and VN.

**Conclusion:**

Serum levels SA, hs-CRP, VN, PAI-1, OX-LDL and MDA may be predictive of adverse cardiovascular outcomes. Interestingly, these analyses can help as diagnostic and monitoring markers in CAD patients.

## Introduction

Atherosclerotic coronary artery disease (CAD) remains one of the world’s major health problems, accounting for 12.7% of global mortality.^1^ As we know, atherosclerosis is known as a chronic inflammatory process that is initiated with the dysfunction or activation of the arterial endothelium. Moreover, endothelial damage and reactive oxygen species (and other free radicals) have emerged as main factors in practically all pathways that lead to the development of atherosclerosis.^2^ Risk factors identified recently that are related to pro-atherogenic cardiovascular disease include those associated with impaired coagulation or fibrinolysis, cardiovascular remodeling and inflammation.^3^ Notably, increase in plasma levels of risk markers for atherosclerotic cardiovascular disease have been recognized to play an important role in both the onset and the progression of atherosclerotic plaque. These prognostic markers may assist in therapy to match the intensity of the patient’s disease.^4-8^

Remarkably, Vitronectin (VN) is present in plasma, extracellular matrix, and granules of blood platelets. It consists of adhesive glycoproteins, which play a key role in the regulation of processes such as platelet adhesion, aggregation and clotting, via binding to integrin, plasminogen activator inhibitor (PAI-1), urokinase plasminogen activator receptor (UPAR), and heparin.^9,10^ In spite of that fact, plasma VN levels were significantly increased in patients with CAD, also showing a positive correlation with severity of the disease.^11^ Notably, PAI-1 has been recognized as a central molecule linked to pathogenesis and progression of thrombotic vascular events, including stroke. In addition, elevated plasma PAI-1 levels are associated with vascular thrombosis.^12^ A previous study suggested that high levels of PAI-1 in CAD are associated with the risk of endothelial dysfunction and premature atherosclerosis.^13^ Sialic acid (SA) is derivative of neuraminic acid, and comprises the terminal sugar part of the oligosaccharide chain in glycolipids and glycoproteins, acting as a cofactor in several cell surface receptors, such as LDL receptor. Its intake of LDL occurs prominently in smooth muscle cells of blood vessels and is increased in several pathological and inflammatory states, such as in atherosclerosis.^5,14,15^ Therefore, following an inflammatory reaction or injury, desquamating or secretion from damaged cells can lead to an elevated concentration of SA.^16^ Oxidative stress and inflammation also play vital roles in the pathogenesis and progression of CAD.^6^ Oxidized low density lipoprotein (OX-LDL) and correlated composites are also observed in lesion formation at the later stages of atherosclerosis. Hence, OX-LDL could play a major role in both atherogenesis and plaque complications.^17,18^ Additionally, malonaldehyde (MDA) results from lipid peroxidation, and its measurement is an undependable marker of oxidative damage, making MDA a suitable indicator and marker for identification and further evaluation of patients with CAD.^17^ Among several markers of inflammation, highly sensitive C-reactive protein (hs-CRP) has been established as significant in people with CAD. Several studies demonstrated that hs-CRP is associated with increased CAD risk.^19^ Previous findings reported elevated VN, MDA, OX-LDL, PAI-1, hs-CRP and SA levels, which were positively correlated with CAD. Although the pathological aspects of these risk factors have been studied, their role has not been recognized in the early and accurate diagnosis of atherosclerosis in patients with CAD. This study aimed to determine concentrations of the prognostic value of serum levels of hs-CRP, SA, VN, PAI-1, OX-LDL and MDA in patients with CAD, all of which can manifest in CAD, in an effort to examine the importance of combining these biomarkers with the diagnosis of CAD.

## Methods

### Subjects

The sample size was 180 subjects, based on a convenience sample. The subjects were divided into 4 groups according to angiography results. The control group was a no Stenosis group which included 40 subjects with non-significant disease that had no clogged vessels but suffered from chest pain such as angina pectoris; 40 with single occluded vessel disease (1VD); 40 with double occluded vessel disease (2VD); and 40 individuals with triple occluded vessel disease (3VD). In addition, the control group was composed of healthy individuals without any presentation of CAD (n = 20). Peripheral blood sampling was obtained after one night of fasting in the Shahid Madani hospital, located in East Azerbaijan, Iran.

### Ethics

Before the beginning of the study, the protocol was presented to the independent ethics committee of the Medical Faculty of the Tabriz University of Medical Sciences (ethics number 91/2-3/5). An informed consent was obtained from all of participants. All patients with the renal disease, lung disorders, liver dysfunction, autoimmune disease, infectious diseases and cancer were excluded from the study.

### Laboratory methods

All blood samples were obtained from a peripheral vein after 12 hours of overnight fasting. Subsequent plasma and serum were separated within 30 minutes, and samples were stored at -70°C until the tests were performed.

### Measurement of parameters

Enzyme-linked immunosorbent assay (ELISA) procedures were used to determine serum levels of OX-LDL (Glory Science co. Ltd, Cat. No: 93614), PAI-1 (Boster Science co. Ltd, Cat. No: EK0859) and VN (Glory Science co. Ltd, Cat. No: 11668). SA was also measured by ELISA, using a commercial kit (Crystal Day, China). Serum MDA was measured based on reaction with Thiobarbituric Acid (TBA); extraction accompanied with normal butanol; absorption measured by spectrophotometer and value calculated according to a standard curve. Serum levels of hs-CRP were estimated by high-sensitivity turbidimetry method using Biosystems kit (Barcelona, Spain, COD 31927); the assay was evaluated on semi-autoanalyser (Alcyon 300, made in USA) in the Biochemistry lab.

### Analytical methods

All statistical analyses were carried out using SPSS software, version 20.0 (SPSS Inc., Illinois, USA). All quantitative variables were expressed as mean ± standard deviation or median and interquartile range. The qualitative variables were expressed in numbers and percentage. The normality of the data was evaluated by the normal curve (skewness and standard deviation of skewness) of Kolmogorov-Smirnov test. Differences for multiple groups were analyzed using independent t-test, and one-way analysis of variance (ANOVA). Also, the chi-square test was used for categorical variables. The Kruskal-Wallis test was used for the quantitative variables if the normality assumption of the residuals was not met. Moreover, ROC curve analysis was used to evaluate the diagnostic effect of VN, MDA, OX-LDL, PAI-1, hs-CRP and SA by logistic regression model. A statistical analysis was defined when p < 0.05.

## Results

**Figure 1:**
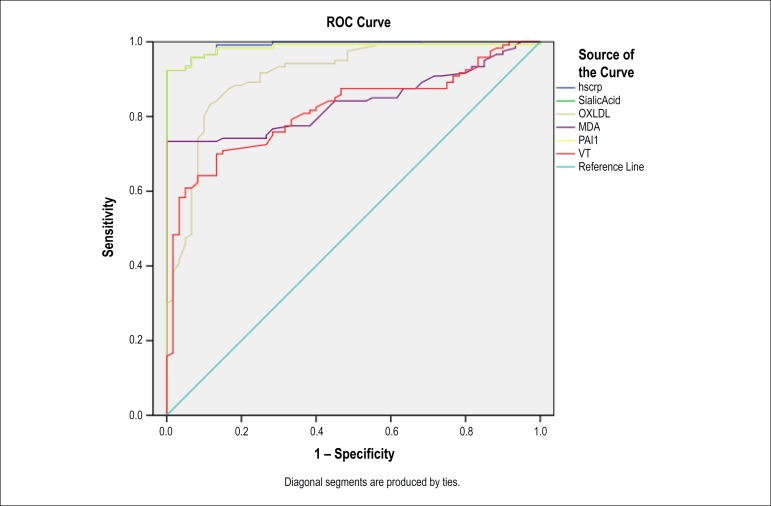
ROC analysis of VN, MDA, OX-LDL, PAI-1, hs‑CRP and SA. VN: vitronectin; MDA: malondialdehyde; OX-LDL: oxidized low density lipoprotein; PAI-1: plasminogen activator inhibitor-1; hs‑CRP: high-sensitivity C‑reactive protein; SA: sialic acid.

**Figure 2:**
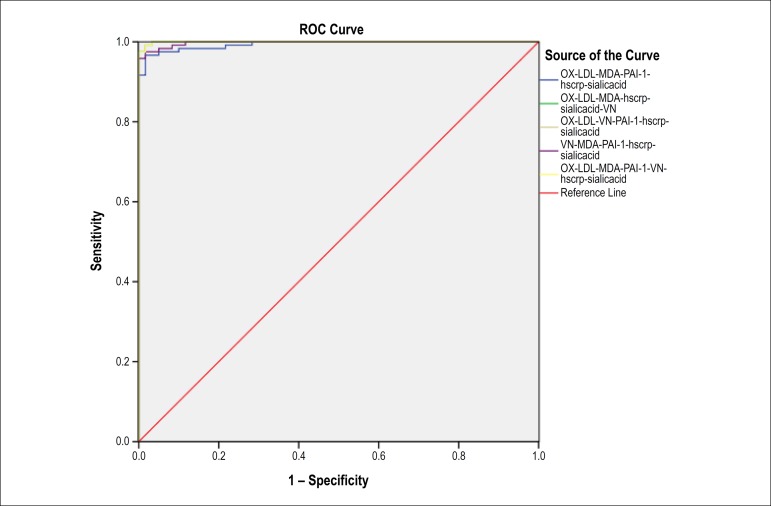
ROC curve of combined assay. VN: vitronectin; MDA: malondialdehyde; OX-LDL: oxidized low density lipoprotein; PAI-1: plasminogen activator inhibitor-1; hs‑CRP: high-sensitivity C‑reactive protein; SA: sialic acid.

The prognostic indicators were used in the current study. Table 1 lists the general characteristics of the study groups. Differences between patient and control groups based on age and sex distribution were subject to an independent-samples t-test. The results showed that there were no significant differences between the groups (p < 0.3). However, smoking, hypertension and diabetes showed significant difference between the patient and control groups (p value: 0.004, 0.01, and 0.02, respectively). Table 2 represents group mean parameter values obtained from a one-way ANOVA analysis; the mean serum levels of parameters that have been compared among subgroups were categorized based on number of occluded vessels of study population. Significant differences in all cardiovascular risk factors measured were found in all subgroups compared to controls (p < 0.001 for all of them). Moreover, there were significant differences among subgroups that were not mentioned (previously reported in other references).^11,20,21^ The critical values of VN, MDA, OX-LDL, PAI-1, hs-CRP and SA levels were determined by ROC curve analysis. Both specificity and sensitivity of these six parameters in CAD were compared. A combined assay was then performed, using six indexes. In addition, using both healthy individuals and CAD patients as subjects, ROC analysis was performed, which showed the areas under the curve for OX-LDL, MDA, PAI, VN, hs-CRP and SA (0.870, 0.804, 0.951, 0.799, 0.962 and 0.971, respectively). All risk factors had satisfactory diagnostic efficacy for CAD. The overall rank of efficacy was (from lower to higher): VN, MDA, OX-LDL, PAI-1, SA and hs-CRP. PAI-1, SA and hs-CRP had particularly high validity in the diagnosis of CAD (Figure 1 and Table 3). Notably, the criteria of variables were specified with reference to appearance levels in both healthy individuals and CAD patients, Cutoff for OX-LDL, MDA, PAI1-, VN, hs-CRP and SA were 2.67 (ug/mL), 5.49 (mmol/mL), 67 (ng/mL), 254 (ng/mL), 3.4 (mg/dL), 7/89 (mg/dL), respectively. The sensitivity and specificity were 70% and 75%, 74% and 77 %, 92% and 90%, 70% and 83%, 94% and 93%, 94% and 96%, respectively. PAI1-, SA and hs-CRP had the highest sensitivity and specificity in the test, compared to OX-LDL, MDA and VN (Table 4). The efficacy of the combined assay was then compared using six parameters (OX-LDL, MDA, VN, PAI1-, SA and hs-CRP). Such combined assay increased the predictive value of sensitivity and specificity to 99% and 99%, respectively (Table 5). The area under ROC curve was 0.99 (95% CI: 0.975~1.005, Figure 2).

**Table 1:** General characteristics of the study groups

Characteristic		Control	No Stenosis	1VD	2VD	3VD	p value
Sample size		20	40	40	40	40	-
Age(mean(SD))		57.5(3.2)	58.80(7.5)	58.9(7.9)	61.0(11.8)	60.5(10.5)	0.37*
Sex(male/female)		17/3	21/19	31/9	30/10	25/15	0.30**
Smoking (N (%))		0(0%)	13(32.5)	19(47.5)	16(40)	24(60)	0.004**
Hypertension (N (%))		0(0%)	25(62.5)	26(65)	12(30)	19(47.5)	0.01**
Diabetes (N (%))		0(0%)	7(17.5%)	11(27.5%)	12(30%)	15(37.5%)	0.02**

* One-way analysis of variance;

** Chi square test.

**Table 2:** Comparison of the mean serum levels of cardiovascular risk factors among subgroups categorized based on number of occluded vessels of CAD patients

p Value	3VD	2VD	1VD	No Stenosis	Control	Variable
0.001^*^	3.13 ± 0.42	2.76 ± 0.38	2.62 ± 0.27	2.28 ± 0.32	1.41 ± 0.22	OX-LDL (ug/mL)^1^
0.001*	7.12 ± 1.21	6.39 ± 0.66	5.25 ± 0.98	5.20 ± 0.44	4.32 ± 0.86	MDA (mmol/mL)^1^
0.001*	86.8 ± 6.8	76.9 ± 4.7	75 ± 14.2	51.5 ± 10.8	41.7 ± 11.9	PAI-1 (ng/mL)^1^
0.001**	361 (95.75)	264 (100.75)	304 (184.25)	208 (61.75)	200 (26)	VT (ng/mL)^2^
0.001**	5.23 (1.05)	7.53 (0.86)	5.21 (0.39)	1.52 (1.03)	2.54 (0.78)	hs-CRP (mg/dL)^2^
0.001*	169.9 ± 15.3	138.3 ± 12.3	108.6 ± 9.2	60 ± 11.6	51.0 ± 5.0	SA (mg/dL)^1^

OX-LDL: oxidation of low-density lipoprotein; MDA: Malondialdehyde; PAI-1: Plasminogen Activator Inhibitor; VT: Vitronectin; hs-CRP: high-sensitivity C-reactive protein; SA: sialic acid; 1VD: Stenosis in one of vessels; 2VD: Stenosis in two of vessels; 3VD: Stenosis in three of vessel.

1 Mean ± standard deviation;

2 Median (inter quartile range).

* Performed by ANOVA test.

** Performed by Kruskal-Wallis test.

**Table 3:** Area under curve of ROC

Test Variable	Area	SD^a^	p value^b^	Asymptotic 95% Confidence Interval	
				Lower bound	Upper bound
OX-LDL	0.870	0.032	.000	0.806	0.934
MDA	0.804	0.034	.000	0.738	0.869
PAI-1	0.951	0.010	.000	0.921	0.992
VT	0.799	0.036	.000	0.729	0.869
SA	0.962	0.021	.000	0.921	0.998
hs-CRP	0.971	0.032	.000	0.953	0.995

Note: ^a^. Under the nonparametric assumption, ^b^. Null hypothesis: true area = 0. OX-LDL: oxidation of low-density lipoprotein; MDA: Malondialdehyde; PAI-1: Plasminogen Activator Inhibitor; VT: Vitronectin; SA: sialic acid; hs-CRP: high-sensitivity C-reactive protein.

**Table 4:** Diagnostic efficacy of parameters

index	Positive criteria	Sensitivity/%	Specificity/%	False negative /%	False positive/%
SA	≥ 89.7 (mg/dL)	94	96	6	4
hs-CRP	≥ 3.4 (mg/dL)	94	93	6	7
PAI-1	≥ 67 (ng/mL)	92	90	8	10
VT	≥ 254 (ng/mL)	70	83	30	17
OX-LDL	≥ 2.67 (ug/mL)	70	75	30	25
MDA	≥ 5.49 (mmol/mL)	74	77	26	23

OX-LDL: oxidation of low-density lipoprotein; MDA: Malondialdehyde; PAI-1: Plasminogen Activator Inhibitor; VT: Vitronectin; SA: sialic acid; hs-CRP: high-sensitivity C-reactive protein.

**Table 5:** Combined assay of PAI-1, VT, OX-LDL, MDA, SA and hs-CRP

Index		Sensitivity/%	Specificity/%
OX-LDL, MDA, PAI-1, SA, hs-CRP		97	95
OX-LDL, MDA, SA, hs-CRP, VN		98	97
OX-LDL, VT, PAI-1, SA, hs-CRP		98	97
VT, MDA, PAI-1, SA, hs-CRP		97	97
OX-LDL, MDA, PAI-1, VT, SA, hs-CRP		99	99

OX-LDL: oxidation of low-density lipoprotein; MDA: Malondialdehyde; PAI-1: Plasminogen Activator Inhibitor; VT: Vitronectin; SA: sialic acid; hs-CRP: high-sensitivity C-reactive protein.

## Discussion

Commonly, major risk factors causing atherosclerotic lesions in human coronary arteries comprise genetic factors, hyperlipidemia, diabetes, infections, hypertension or oxidative stress, with little correlation with patients’ age and environmental factors.^22^ It is noteworthy that this pathological process includes macrophages and smooth muscle cells (SMCs), with addition and deposition of lipids and extracellular matrix proteins, especially glycoprotein.^22,23^ Previous studies confirmed that serum levels of VN, PAI-1, OX-LDL, MDA, hs-CRP and SA are significantly higher in CAD patients compared with healthy controls and positive correlation with severe diseases. A climb in these analyses in patients with CAD when compared to controls is already well distinguished in recent studies.^11,20,21,24^ The present study considerably assessed the prognostic value of glycoprotein, fibrinolysis, oxidative stress and inflammatory biomarkers including VN, PAI-1, and OX-LDL, MDA, hs-CRP and SA in patients with CAD.

Remarkably, VN is a multifunctional plasma glycoprotein with a multiple binding domain, which regulates processes such as platelet adhesion, aggregation and clotting. Besides, VN can be expressed and produced in the vessel wall, predominantly in atherosclerotic lesions.^25^ Later studies showed that PAI-1 stimulates VN expression in SMCs by binding LDL receptor-related protein-1 (LRP1), and controls vascular VN expression in vivo. Therefore, autocrine regulation of vascular VN expression by PAI-1 may play important roles in vascular homeostasis and pathologic vascular remodeling.^26^ Several studies have found a regulatory function for VN in the hemostatic response to vascular injury.^9^ Also, VN binds PAI-1 and adjusts its action by stabilizing the active PAI-1 conformation, and potentially controls PAI-1 clearance.^27^ Serum levels of VN were found to be increased patients with CAD when compared with controls.^9,11^ Derer et al.^26^ suggested that VN is a clinically useful biomarker for unfavorable cardiovascular outcomes in patients following acute stenting undergoing coronary interventions.^26^ Therefore, VN may serve as a marker for CAD, and elevated levels may indicate its role in the diagnosis and/or progression of CAD. Notably, there is suggestion that high plasma PAI-1 concentrations are related with the progression of coronary syndromes and the development of myocardial infarction.^26,28^ Clinical and experimental studies demonstrated that PAI-1 deficiency in humans is accompanied by abnormal bleeding, whereas elevated PAI-1 plasma levels are associated with vascular thrombosis, indicating the crucial role of PAI-1 in hemostatic clot stabilization.^13^ Moreover, previous studies have shown that PAI-1 is significantly elevated in CAD patients in comparison to controls, and it has also a significant relationship with severity of the disease.^20,29^ In addition, it was reported that PAI-1 is an independent predictor of coronary microvascular dysfunction in hypertension.^30^ Our results suggested that prominent levels of PAI-1 concentrations may predict and be a diagnosis marker for CAD.

Furthermore, oxidative stress has an important role in the beginning and progression of atherosclerosis. OX-LDL is more atherogenic than the native LDL, and has been recognized to accumulate in atherosclerotic lesions in the aorta and coronary arteries of patients with CAD.^2,8,17^ Additionally, MDA is produced from breakdown of lipids during peroxidation processes, and serum MDA is a reliable marker of oxidative damages. Previous findings also have confirmed the involvement of lipid peroxidation in CAD by referring to the plasma levels of MDA observed in CAD patients compared with healthy controls. More recent cross-sectional studies demonstrated a positive relationship between elevated levels of OX-LDL and MDA with severity of acute coronary syndromes.^20,31^ Ehara et al.^32^ reported that the plasma OX-LDL level in patients with CAD increases by approximately 3.5 fold than in control subjects.^32^ Remarkably, the findings in this study indicated that both oxidative stress parameters can be used as diagnosis markers of CAD, and the impact of this oxidative stress may progress to an atherosclerotic event. Impressively, in arterial injury accompanied by inflammatory response, inflammation plays a key role in the pathogenesis of CAD and its impediments. Hence, SA and hs-CRP have gained importance as inflammatory markers and as indicators and predictors of the process of acute coronary syndromes.^19,33^ Increased production of isolated acute phase reactants increases the SA levels. SA is associated with atherosclerosis independently of other cardiovascular risk factors.^15^ Previous studies have reported total serum levels of SA that were high in patients with acute coronary syndrome when compared to healthy controls.^34^ Specifically, Govindarajan et al.^14^ showed that the total plasma SA level was significantly higher in patients with myocardial infarction than in those with unstable and stable angina. In a recent 17-year follow-up study, elevated serum levels of SA were found to be predictive of cardiovascular events in apparently healthy individuals.^35^ In addition, several studies suggested a positive relation between hs-CRP and CAD among healthy individuals.^36,37^ Mahajan et al.^38^ found a relation between inflammatory markers and coronary artery involvement on diabetic patients suffering from early onset CAD.^38^ In addition, many evidences have indicated that hs-CRP is a cautiously sensitive systemic marker for diagnosis of inflammation and a useful and potent predictive marker of cardiovascular events.^11,39^ This study has shown that serum hs-CRP and SA levels may be used as predictive or diagnosis biomarkers in patients with CAD.

The findings in this study showed significant elevated serum levels of OX-LDL, MDA, PAI1-, VN, hs-CRP and SA in patients with CAD, when compared to healthy individuals. hs-CRP, SA and PAI-1 had the best sensitivity and specificity, suggesting the value of these biomarkers in patients with CAD diagnosis. ROC curve analysis showed satisfactory diagnostic power of all these six indexes, from highest to lowest: SA, hs-CRP, PAI-1, OX-LDL, MDA, and VN.

This study also considered the diagnostic value of combined assay for all indexes for the best confirmative diagnosis value, including hs-CRP, SA, PAI-1, OX-LDL, MDA, and VN, resulting in elevated sensitivity and specificity values, without significant decrease of negative predictive value. These results supported the complementary role of combined assay in diagnosis of CAD.

One of limitations of this study is the probability of a non-representative sample, in an attempt to select a random sample, since the hospital is a referral hospital and the patients approached this hospital in special days, which may lead to selection bias.

Also, this study is only cross-sectional, which might not show temporal relationships, and thus the observed associations may not necessarily be causal.

## Conclusions

In the present study, high serum concentrations of SA (≥7/89 mg/dL), hs-CRP (≥3.4 mg/dL), PAI-1 (≥67 ng/mL) and increase in OX-LDL, MDA, and VN were found to be independent significant predictors of CAD in patients. In addition, the results suggested that using serum levels of hs-CRP, SA, PAI-1, OX-LDL, MDA, and VN may be helpful in clinical monitoring. The combined assay of serum PAI-1, OX-LDL, MDA, and VN can improve the sensitivity and specificity for diagnosing CAD, and can be used for population screening and for monitoring patients with CAD. Therefore, while we suggest the use of these biomarkers as a diagnostic apparatus for CAD patients, they need to be further explored to confirm this suggestion.
